# Openness and productivity of the Swiss economy

**DOI:** 10.1186/s41937-018-0021-3

**Published:** 2018-09-17

**Authors:** Reto Föllmi, Angela Fuest, Philipp an de Meulen, Martin Micheli, Torsten Schmidt, Lina Zwick

**Affiliations:** 1 SIAW-HSG, Universität St. Gallen, Bodanstrasse 8, 9000 St. Gallen, Switzerland; 2 RWI, Leibniz Institute for Economic Research, Hohenzollernstr. 1-3, 45128 Essen, Germany; 3 FOM Hochschule für Oekonomie & Management gGmbH, Campus Dortmund, Lissaboner Allee 7, 44269 Dortmund, Germany

**Keywords:** Productivity, Openness, Trade barriers, O40, F10, F30

## Abstract

This paper analyzes the connection between openness and economic performance in Switzerland. Considering different dimensions of openness, we show that the Swiss economy is classified as relatively open. Nevertheless, there still is potential to further increase international integration, particularly through deregulation in the services sector. We also show that for some branches in the Swiss manufacturing sector, increases in international trade are associated with higher productivity in the long run. With regard to financial openness, we show that in the aftermath of the financial crisis, Switzerland mainly suffered from capital retrenchment. Foreign capital inflows were of minor importance. Short-run costs due to high volatility of capital flows might therefore be lower than widely perceived.

## Introduction

The growth of labor productivity in Switzerland has been low compared to many other advanced economies in recent decades. In developing economies, productivity growth even improved substantially (OECD 2015a). The acceleration of economic growth in emerging economies came in hand with an integration into global value chains, resulting in increased international trade in goods and services as well as international financial flows. This raises the question of how globalization and productivity are related and why the Swiss economy was not able to benefit so much from these developments.

The process of globalization that started in the mid-1980s not only offers new opportunities but also involves risks for advanced economies. With regard to openness to international trade, taking advantage of comparative advantages and offshoring relatively undemanding jobs to developing economies changes job profiles in advanced economies. However, adapting to globalization might foster productivity growth. The economic literature emphasizes three channels through which international trade might spur productivity. First, the international trade increases the competition on domestic markets and forces domestic firms to raise productivity (Melitz 2003). Second, it supports the diffusion of knowledge (Grossman and Helpman 2015), and third, it raises market sizes that allow for economies of scale (e.g., Alesina et al. 2005). Empirical studies support the positive relation between trade openness and productivity growth (Edwards 1998). However, in order to exploit productivity gains from globalization, advanced economies need to remain innovative and stay ahead of the product cycle (e.g., Foellmi et al. 2018).

With regard to financial openness, free movement of capital allows for a balancing of capital scarcities and surpluses. It should therefore result in a more efficient allocation of capital. Additionally to that, capital flows might affect the production technology, especially if capital inflows are connected to foreign investments. Kose et al. (2009b) show this positive effect of financial openness on productivity growth empirically. However, the authors only find evidence for a positive relation between de jure financial openness and productivity. The link between de facto financial openness and productivity is less clear. A drawback of financial openness is a short-run downside risk, even for advanced economies with highly developed financial markets, due to the high volatility of financial flows.

In this paper, we explore the connection between international interconnectedness, defined as trade and financial openness, and productivity in Switzerland. While empirical studies mainly use cross-country-, industry-, or firm-level data (Hufbauer and Lu 2016), studies at the country level only exist for catching up economies (e.g., Kappeler 2015). Switzerland is interesting to study as it is a highly innovative industrialized economy. Studying the link between trade openness and productivity in an advanced economy might therefore yield important insights. Given Switzerland’s already high productivity, another interesting question is whether capital inflows from other highly developed economies are still associated with increases in economic activity. A further question of interest regards potential costs of Switzerland’s financial openness. On the one hand, Switzerland has a highly developed financial sector and has not been directly affected by the European debt crisis. On the other hand, Switzerland persistently runs current account surpluses such that a global economic slowdown should result in a capital retrenchment. However, the cause of net capital inflows has substantially different implications for the costs of and therefore the desirability of financial openness. This makes Switzerland an especially interesting country to study.

In the “International trade and financial flows: the openness of the Swiss economy” section, we consider different dimensions of openness and give our assessment of the Swiss economy’s openness. The “Trade openness of the Swiss economy employing the OECD approach” section discusses the difference between our assessment and the OECD’s diagnosis of Switzerland being a relatively closed economy (OECD 2013b). The “The link between international trade and productivity” section analyzes the long-run relation between trade and financial openness and labor productivity in Switzerland at the aggregate level as well as for the different branches of the Swiss manufacturing industry. In the “Regulation of trade in services” section, we simulate the effect of deregulation in the Swiss services sector. The “Switzerland and the cyclicality of capital flows” section analyzes the relation between international capital flows and economic activity in Switzerland and discusses potential short-term costs due to the high volatility of capital flows. The “Conclusions” section concludes.

## International trade and financial flows: the openness of the Swiss economy

The assessment of an economy’s openness to international markets is not a trivial task. This is mainly due to the fact that there are different definitions of openness. First, there is a distinction between de jure and de facto openness. De jure openness refers to the degree of political restrictions imposed on international trade flows, while de facto openness refers to an economy’s degree of integration into international markets based on actual flows. Second, there are different types of flows, i.e., financial flows and flows in goods and services. In the following, we compare different measures of openness to provide a broad picture of the openness of the Swiss economy.

**Fig. 1:**
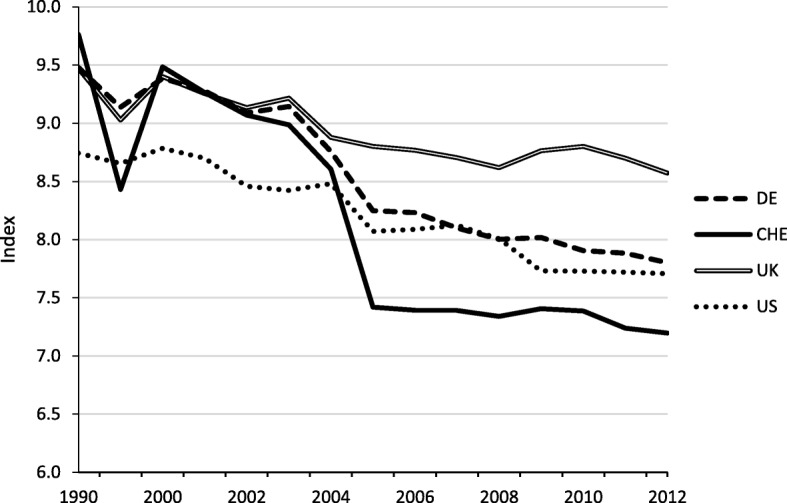
Freedom to trade internationally. Sample: 1990–2012, Source: Fraser Institute

First, we consider de jure openness. According to the Economic Freedom Index released by the Fraser Institute, trade restrictions imposed by tariffs and non-tariff trade barriers as well as capital controls have intensified since 2000. Although the exact number must be interpreted with care, the index for Switzerland decreased markedly in 2004 and 2005. It has been considerably lower for Switzerland than for similar countries ever since (Fig. 1).^1^ The Global Competitiveness Index, published by the World Economic Forum, supports this picture of relatively high trade restrictions in Switzerland. With respect to trade barriers, Switzerland ranked 114th among 151 countries in 2013/2014. International trade therefore seems to be impeded by comparably high tariffs and other trade barriers.

In addition, the Swiss regulatory environment is not conducive to business operation. According to the World Bank’s Ease of Doing Business Index from 2016, Switzerland’s business climate was affected, e.g., by inefficiencies with regard to starting a business, dealing with construction permits, and getting credit.

Despite these shortcomings, Switzerland is characterized by sound institutional quality and a favorable investment climate. According to the Global Competitiveness Index, investors’ property rights are strongly protected and investment incentives are barely distorted by taxation. Taken together, in terms of de jure openness, the Swiss Economy may still be labeled as relatively open, even though Switzerland’s openness has slightly decreased in recent years.

**Fig. 2:**
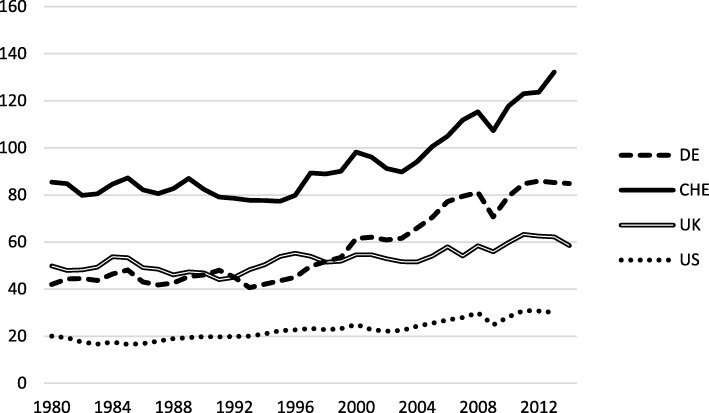
Nominal Openness. In percent. Sample: 1980–2014, Source: International Trade Database, OECD

A major problem with interpreting de jure measures is that they are usually based on an ordinal scale. De facto openness, measured by actual flows of capital and goods, is thus more meaningful. The most common measure for a country’s de facto openness is nominal trade openness defined as the sum of nominal exports and imports divided by nominal GDP. Between 1980 and 1995, Switzerland’s external trade grew less than its GDP. In the subsequent years, however, nominal openness increased considerably. In contrast to many similar countries, Switzerland’s nominal openness increased considerably in the years following the Great Recession (Fig. 2). Therefore, compared with other countries, Switzerland may now be labeled as a de facto very open economy. This holds for both trade in goods and trade in services. Nevertheless, trade openness in the services sector has not increased since 2000, whereas external trade in goods has grown from 70 to over 100% in relation to GDP.

It should be noted that there are also some drawbacks to using *nominal openness* to assess the openness of an economy. Alcala and Ciccone (2004) argue that an increase in external trade may lead trading partners to increase specialization according to their comparative advantage. Because of specialization, formerly domestically produced goods are substituted by imports, which increases productivity in the tradable goods sector but not in the non-tradable goods sector. Due to this asymmetry, the relative cost of production in the non-tradable goods sector increases, which results in a rise of relative prices and hence a rise of nominal GDP. This will increase the size of both denominator and numerator of the nominal openness measure. Thus, an expansion of international trade might decrease nominal openness according to this measure.

**Fig. 3:**
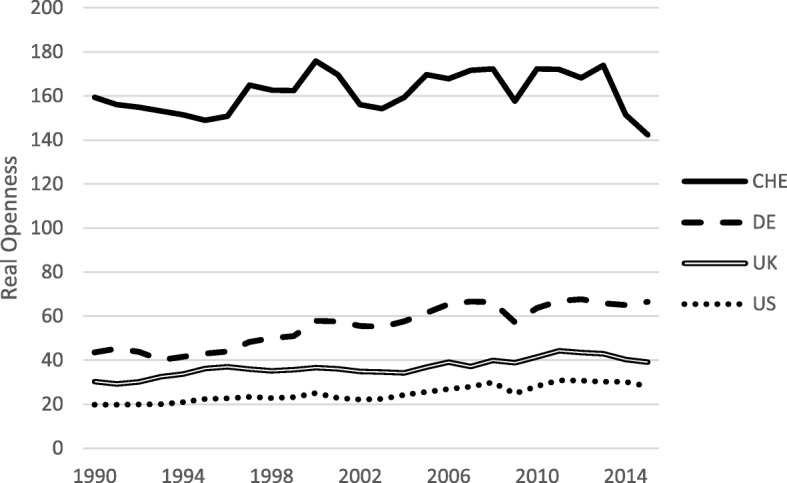
Real Openness. Sample: 1990–2015, Source: International Trade Database, OECD

As an alternative, Alcala and Ciccone (2004) propose a measure that assesses the so-called real openness by using GDP based on purchasing power parity. This measure identifies Switzerland evidently as an open economy (Fig. 3). However, real openness also reflects international differences in purchasing power. Due to Switzerland being a highly productive economy, the price level in the non-tradable goods sector is extraordinarily high in international comparison. Therefore, GDP based on purchasing power parity is substantially lower, resulting in a higher degree of openness according to the real openness indicator.

**Fig. 4:**
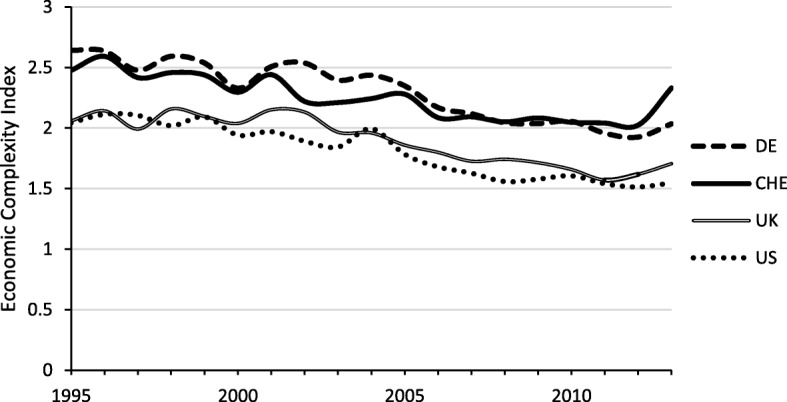
Complexity of exported goods. Sample: 1995–2013, Source: Economic Complexity Index

Another measure of an economy’s de facto openness is the Economic Complexity Index constructed by Hausmann and Hidalgo (2014). This index includes information on the diversity of traded goods and on the number of countries that are able to produce and supply these goods.^2^ Figure 4 illustrates that the export structure of the Swiss economy is relatively complex and diversified. Switzerland has been ranked among the top five countries on the Economic Complexity Index since its introduction in 1995. According to Hausmann and Hidalgo (2014), a larger variety and higher complexity of exported products is associated with higher growth, even in the short run (Hausmann and Hidalgo 2009, 2014).

Besides trade openness, a country’s openness can also be defined in terms of its openness to financial flows. Financial openness can again be measured in terms of de jure and de facto openness. Regarding the former, Switzerland’s financial markets are barely regulated. There are neither noteworthy barriers to entry nor restrictions regarding the pricing and volume of credit (Abiad et al. 2010).

**Fig. 5:**
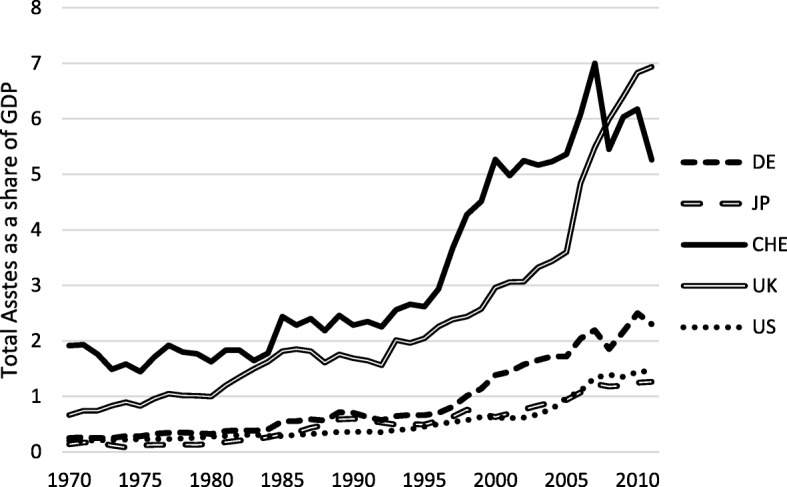
Gross foreign assets as a share of GDP. Sample: 1970–2011, Source: External Wealth of Nations, Lane and Milesi-Ferretti (2007), updated dataset

**Fig. 6:**
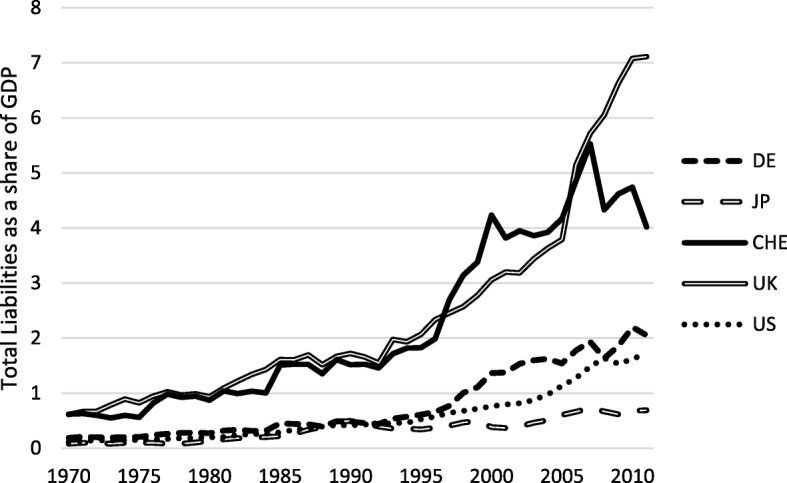
Gross foreign liabilities as a share of GDP. Sample: 1970–2011, Source: External Wealth of Nations, Lane and Milesi-Ferretti (2007), updated dataset

De facto integration on international financial markets may be assessed by measures of openness to foreign investment, i.e., via portfolio or direct investment (Kose et al. 2009a, b). Figures 5 and 6 illustrate the resulting stock values of gross foreign assets and gross foreign liabilities, respectively, for selected countries, based on the “External Wealth of Nations” dataset by Lane and Milesi-Ferretti (2007). High assists relative to GDP indicate that a country is a target for international investors; high liabilities show that it is easy to transfer funds to foreign countries. Switzerland’s de facto financial openness therefore seems to be quite high.

In the following, we want to explore whether the high degree of financial openness in Switzerland might affect productivity growth. The literature assumes that different forms of capital investment have different effects on economic growth.

One type of capital flow that potentially promotes productivity gains is Foreign Direct Investment (FDI). FDI can entail a transfer of technological and management know-how. Moreover, foreign direct investment decisions are much more long-term oriented than credit flows and thus facilitate knowledge spillovers to other enterprises and sectors.^3^

**Fig. 7:**
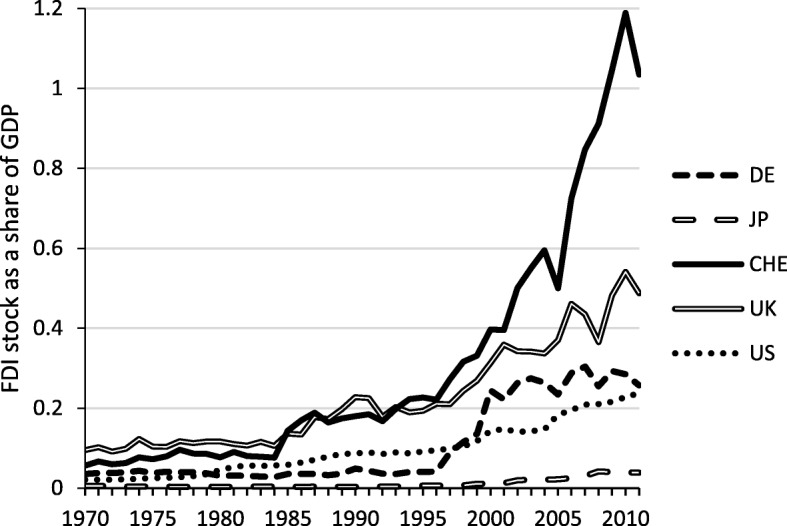
Stock of FDI as a share of GDP. Sample: 1970–2011, Source: External Wealth of Nations, Lane and Milesi-Ferretti (2007), updated dataset

As the quality of imported knowledge depends on the country of origin, it is worth noting that 98% of Switzerland’s FDI inflows originate from Europe and the USA and 68% from Germany, France, Luxembourg, the Netherlands, and Austria, all of which are technologically advanced countries. The largest share of FDI inflows was received by the services sector. In 2013, the industrial sector accounted for around CHF 100 bn of the value of the FDI stock. In the services sector, the value was nearly CHF 600 bn. FDI inflows to the service sector as share of total FDI thereby exceeded the sectors’ share in the economy. Within the services sector, the stock of FDI in the generally knowledge-intensive finance and holding companies was worth CHF 358 bn (SNB, 2014). Compared to Germany, Japan, UK, and the US, Switzerland is characterized by a very large GDP share of the stock of inflowing FDI (Fig. 7). This likely represents the fact that Switzerland is an attractive and international financial center. However, recently, Switzerland suffered considerable withdrawals of capital, in particular from several finance and holding companies (SNB 2014).

In sum, Switzerland is already a relatively open economy. This assessment holds true for de facto openness measures, particularly with regard to financial flows, which are based on actual data. This is also confirmed by Weder (2013), who states that among industrial economies, Switzerland’s trade integration is above average, and by Dümmler (2016), who reports that in 2015, “70% of (Switzerland’s) GDP [ … ] was earned abroad”. Following the KOF Index of Globalization, Switzerland is one of the most economically globalized countries in Europe, with regard to actual flows. At the same time, however, it is not as globalized as other European countries. Hence, as a means to also improve de jure openness, Switzerland may consider deregulation and a reduction of trade restrictions on goods and services.

## Trade openness of the Swiss economy employing the OECD approach

In contrast to our assessment of Switzerland’s openness, the OECD labeled Switzerland as a relatively closed economy (OECD 2013b). This assessment, however, is not to be understood in absolute terms. Measured by international trade volumes, the Swiss economy is by no means a closed economy. However, given the small population size and its geographic location—Switzerland is surrounded by economically potent neighbors—Swiss’ trade volumes could be expected to be even higher. The OECD concludes that Swiss politics should facilitate international trade to exploit the economy’s maximum potential (OECD 2013b, 72).

The OECD uses a cross section of OECD countries to estimate a gravity-type model, which is typically used to explain trade flows. Trade openness—measured by foreign trade in relation to economic activity—is explained by two variables: foreign demand for domestic products and the size of the domestic economy. The domestic economy’s size, approximated by population size, is expected to have a negative effect on trade openness. The underlying presumption is that production has to exceed a certain threshold in order for an economy to produce competitively on international markets. Therefore, the number of potentially competitive industries in an economy, which lowers the need to import these products, should increase with population size. Foreign demand, on the other hand, should have a positive effect on trade openness. The market for domestic products should increase with rising foreign demand. To approximate foreign demand, the OECD constructs a variable that weights the size of a target market—measured by GDP—by the inverse of the distance to the respective target market.

The OECD uses several different weighting schemes for constructing this demand variable. The first weighting scheme weights a country’s GDP by the inverse of the distance between the two countries’ capitals. Because the importance of a target country as a market for exports might decrease by more than one for one in distance, the OECD employs the inverse of the squared distance between the countries’ capitals as a second weighting scheme. For the third and fourth weighting schemes, the OECD uses data by G-Econ (http://gecon.yale.edu). This dataset contains information on economic activity for 27,000 cells with a grid length of 1° (Nordhaus et al. 2006). The third measure uses economic activity in a cell and weights this activity by the inverse of the distance to the economically weighted Swiss grid cells. The fourth measure uses the inverse of the squared distance as a weighting scheme.^4^

We replicate the OECD results by estimating the following equation:

1 $$ {TO}_j={\beta}_0+{\beta}_1{POP}_j+{\beta}_2{Gravity}_j+{\varepsilon}_j. $$

*TO*_*j*_ is total foreign trade divided by economic activity, our indicator for trade openness of economy *j*, *POP*_*j*_ represents total population, *Gravity*_*j*_ is our proxy for foreign demand for domestic products, *ε*_*j*_ is the error term^5^, and *β*_*i*_ represents the coefficients with *i ϵ {0,1,2}*.

**Fig. 8:**
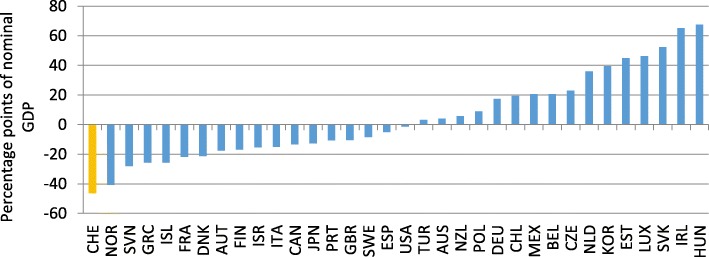
Comparison of estimated and actual trade openness using the inverse of the squared distance between grid cells as weighting scheme for economic activity. Year: 2012, Source: Authors’ calculation based on information by SECO

Based on the Economic Outlook 93 (OECD 2013a), we estimate Eq. (1) for the year 2012 and employ the four demand measures for *Gravity*_*j*_.^6^ The OECD evaluates a country’s openness by calculating the difference between actual trade openness *(exp (TO*_*j*_*))* and expected trade openness according to the estimated coefficients based on the estimated Eq. (1) using the inverse of the squared distance between grid cells as weighting scheme for economic activity (*exp (E (TO*_*j*_*))).* Employing this procedure, Switzerland seems to have substantial backlog with regard to trade liberalization (Fig. 8).

The discrepancy between actual trade openness and the model-based measure of openness for Switzerland is the most pronounced one among all OECD countries.^7^ This, however, does not mean that Switzerland is the most closed economy in our sample. It merely shows that given the geographic circumstances of economically prospering regions nearby—which results in a potentially high foreign demand for Swiss products—and the relatively small size of Switzerland—which prevents Switzerland from specializing in all products that are domestically consumed and therefore makes foreign trade highly desirable—foreign trade in relation to economic activity is relatively low. This discrepancy between actual and projected trade openness is robust to all other demand measures under consideration (OECD 2013b), even though it is most pronounced for the demand measures based on grid data (model 3 and ,model 4). One reason for this might be the proximity of economically prospering regions in Germany (Munich, Stuttgart) as well as in Italy (Milan). Using grid-based weights should therefore increase foreign demand and thereby expected foreign trade in comparison to the calculations based on the inverse of the distance to the capital as a weighting factor.

However, macroeconomic variables are subject to revisions, which can be substantial. In the following, we therefore re-estimate Eq. (1) using the Economic Outlook No. 96 (OECD 2014a). In a first step, we only update the left-hand side variable. Estimation results are reported in Table 1. Coefficients are very similar to the ones using the Economic Outlook No. 93 (OECD 2013a).

**Table 1:** Estimation results, explaining trade openness in 2012

	Model 1	Model 2	Model 3	Model 4
Constant	−2.21* (− 1.76)	2.43*** (3.96)	− 2.74** (− 2.31)	2.45*** (6.69)
Population	− 0.18*** (− 5.30)	− 0.19*** (− 5.45)	−0.19*** (− 5.80)	−0.20*** (− 9.26)
Demand	0.51*** (5.32)		0.57*** (6.14)	
Demand^2^		0.20*** (5.24)		0.24*** (8.76)
Adjusted *R*^2^	0.70	0.70	0.74	0.77
Standard error	0.29	0.30	0.27	0.26

*t* statistics in parentheses; *significant at the 10% level; **significant at the 5% level; ***significant at the 1% level. Demand weighted by the inverse of (squared) distance between capitals in model 1 (model 2), weighted by the inverse of (squared) distance between cells model 3 (model 4)

**Fig. 9:**
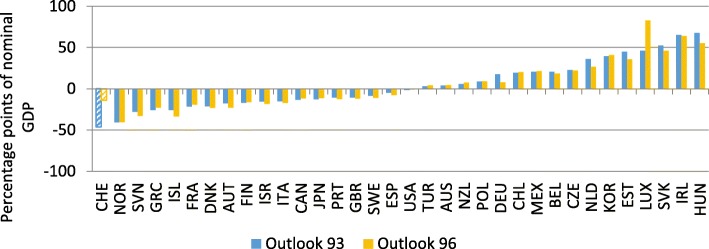
Comparison of estimated and actual trade openness (Model 4). Year: 2012, Source: Authors’ calculation based on information by SECO and OECD (2014a)

However, the assessment of trade openness, given population size and geographical position, has changed substantially for some countries (Fig. 9). Luxembourg and Switzerland seem to be more open. Germany and the Netherlands appear to be much less open.

**Fig. 10:**
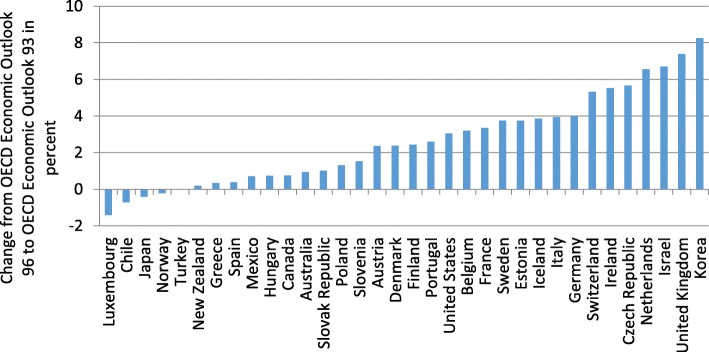
Revisions of GDP. Year: 2012, Source: Authors’ calculations, OECD (2013a), OECD (2014a)

**Fig. 11:**
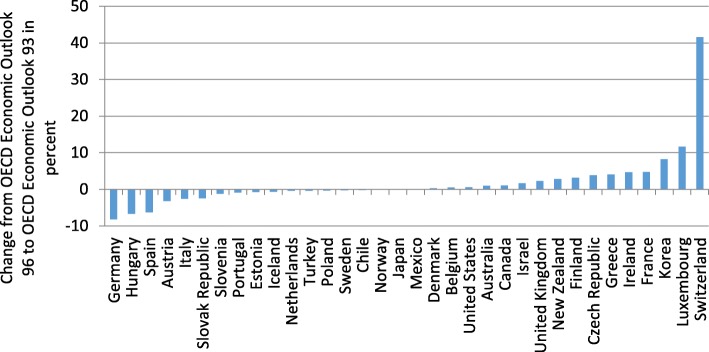
Revisions of foreign trade*.* Year: 2012, Source: Authors’ calculations, OECD (2013a), OECD (2014a)

This change in the assessment of trade openness can be traced back to the switch of the EU accounting framework from The European System of National and Regional Accounts (ESA) 1995 to ESA 2010 in 2014. In particular, foreign trade was subject to redefinitions, which resulted in massive revisions. Changes in GDP have been small in comparison (Figs. 10 and 11).

**Fig. 12:**
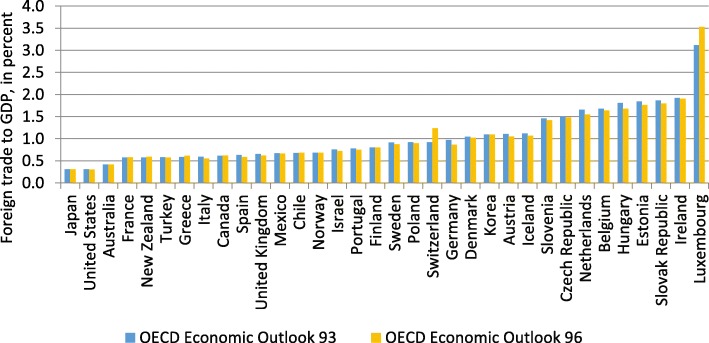
Foreign trade to GDP. Year: 2012, Source: Authors’ calculations, OECD (2013a), OECD (2014a)

In line with the different assessment of trade openness on the basis of the gravity model (Fig. 9), the switch in accounting standards resulted in a substantial increase in our measure for trade openness (Fig. 12). This might be attributed to a change of the definition of transit trade (Federal Statistical Office 2014). In ESA 95, the main criterion for classifying trade of goods had been the physical crossing of borders. An important share of Swiss trade is trade in raw materials which do not actually cross the border. This kind of trade had therefore been classified as trade in services. Under ESA 2010, the main criterion for foreign trade is change in ownership. If one counterpart in the trade of raw materials resides in Switzerland, the transaction is classified as trade in goods. This modification in accounting standards has therefore changed the assessment of the openness of the Swiss economy, which seems to be much less closed than the initial OECD estimates suggest.

In sum, when taking into account the new classification of the national accounts (ESA 2010), the finding that Switzerland—given its small size and the geographic proximity to economically prospering regions—should be classified as relatively closed in comparison to other OECD countries can hardly be maintained. Controlling for these two factors, Switzerland ranks average among OECD countries. However, which accounting standard captures foreign trade more accurately is up for debate.

## The link between international trade and productivity

### Theoretical background

The increasing openness of Switzerland during the last decades raises the question whether and in which sectors the Swiss economy has benefited from this development. The economic literature highlights three channels through which openness can have an impact on the domestic economy: (1) international trade, (2) international capital flows, and (3) international productivity and technological spillovers.

First, the exchange in goods is the key mechanism in the theory on international trade. This theory states that international trade raises productivity because economies are able to specialize on the production of specific products for which they have a comparative advantage. Comparative advantages may result from economies of scale, differences in technology, or resource endowments.

Moreover, international trade should result in increased competition in the tradable goods sector. This should raise productivity in the tradable goods sector as some domestic goods can be substituted by imports. Melitz (2003) argues that an increase in exports may also raise domestic productivity. He shows that international trade fosters reallocation of resources towards more productive firms.

Second, an economy’s openness can also lead to a higher labor productivity by fostering capital accumulation. This is the essence of the financial flow channel. Improved access to international capital (markets) facilitates overcoming domestic capital shortages by capital imports. Hence, a higher capital stock leads to higher labor productivity and stronger economic growth compared to economies in which capital is scarce.

International financial integration also increases domestic incomes if the economy has excess capital. If desired domestic savings are higher than desired domestic investments, this would result in overinvestment in a closed economy, depressing the real interest rate. Financial integration allows for transfers of excess savings to countries where capital is scarce. This raises capital income and thereby gross national income (GNI) compared to the case of a closed economy.

Increasing financial market integration also enhances investment opportunities. Therefore, investors are able to reduce the overall risk of their portfolios. The diversification of idiosyncratic national risks allows for a more efficient allocation of resources and boosts the productivity of inputs (Obstfeld 1994). In addition, a broader diversification of risks reduces risk premiums and hence costs, which positively affects investment demand and productivity (Errunza and Losq 1985).

The third channel through which openness may raise productivity and economic growth is the diffusion of technological knowledge. The theory of innovation-based economic growth shows that monopolistic competition in the sector of intermediate products generates incentives for R&D activities, e.g., to develop new designs for product editions. For this purpose, the prospect of monopoly rents that cover the cost of innovation is essential.

These R&D activities do not only increase product variety but lead to an increase of embodied productivity and improved management techniques (Lunn 1986). As ideas are non-excludable in use, they can spread internationally. Moreover, diffusion of innovations results from international trade of investment and intermediate goods. This process increases productivity in countries in which these new products are used. These spillovers reduce the incentives to invest in R&D activities. However, a larger market may open the opportunity for higher returns and therefore promotes innovations. Furthermore, international trade may raise productivity by increasing the number of international investment goods. However, this occurs only if the elasticity of substitution between these investment goods is small (Rivera-Batiz and Romer 1991; Romer 1990).

### Empirical analysis at the aggregate level

In this section, we analyze the empirical relationship between various measures of openness and labor productivity at the national level. As measures for trade openness, we use nominal openness, real openness, and import and export shares. To measure financial openness, we use the ratio of gross foreign liabilities to GDP. In addition, we use different components of this variable because they may have different effects on labor productivity. For example, FDI has a positive effect on productivity whereas external debt has a negative impact (Kose et al. 2009b). We therefore use FDI foreign liabilities as well as equity capital and borrowed capital in relation to GDP in the following analysis. As a dependent variable, we use labor productivity as well as real GDP based on purchasing power parity from the Penn World Tables. We use annual data from 1980 to 2013.

For this analysis, we estimate several vector autoregression (VAR) models for Switzerland. As the aim of this paper is to present evidence for Switzerland, we employ a time series approach using Swiss data. To take stochastic trends in the data into account, we use the VAR in the error correction representation and test for cointegration between openness and productivity. Similar bivariate approaches have been employed, for example, by Federico et al. (2017) to analyze the link between openness and economic growth and Gunes and Kose (2013) for the link between openness and productivity. Related error correction analysis include additional variables (Malhotra and Kumari 2016; Siliverstovs and Herzer 2006).

We estimate the following bivariate error correction model with one cointegration relation.^8^

2 $$ {\displaystyle \begin{array}{l}\Delta {y}_t={\alpha}_1\left[{y}_{t-1}-\beta {x}_{t-1}-{c}_0\right]+{\delta}_{11}\Delta {y}_{t-1}+\dots +{\delta}_{1k}\Delta {y}_{t-k}+{\gamma}_{11}\Delta {x}_{t-1}+\dots +{\gamma}_{1p}\Delta {x}_{t-p}+{c}_1+{\varepsilon}_{1t}\\ {}\Delta {x}_t={\alpha}_2\left[{y}_{t-1}-\beta {x}_{t-1}-{c}_0\right]+{\delta}_{21}\Delta {y}_{t-1}+\dots +{\delta}_{2k}\Delta {y}_{t-k}+{\gamma}_{21}\Delta {x}_{t-1}+\dots +{\gamma}_{2p}\Delta {x}_{t-p}+{c}_2+{\varepsilon}_{2t},\end{array}} $$

where *y* denotes labor productivity and *x* denotes an explanatory variable—the particular openness indicator. With two variables, there is at most one cointegration vector in this system.

For the interpretation of the results, it is important to distinguish the long-run and the short-run structure of the model (Johansen and Juselius 1994). The long-run structure is based on economic theory. In our case, this is simply the relationship between the level of productivity (*y*) and the level of openness (*x*). Even though both variables exhibit a stochastic trend and are therefore non-stationary, if there is cointegration between the two variables, the residual of the long-run equation is stationary. To facilitate the interpretation, the coefficient for the level of productivity is normalized to one in system (2). The slope coefficient in this long-run relation (*β*) is particularly important as it indicates the co-movement between the level of productivity and the level of openness in Switzerland in the long-run. The constant (*c*_o_) is included in the long-run relationship because there is a trend in the data. The adjustment coefficients *α*_1_ and *α*_2_ indicate through which variable and how rapid the system reverts to its stable long-run relationship.^9^ If a variable does not adjust, it is called weakly exogenous to the system. Hence, these coefficients reveal some information about the causal link between the variables of interest (Hendry 1995). Of course, one has to treat the expression “causal” with care. It is a result of a well-established times-series econometric technique, so it is causation in a time series context. Obviously, this is not the same as causality in a quasi-experimental situation. The results for the adjustment parameters (*α*_1_ and *α*_2_) and the long-run coefficients (*β*) for the relation between openness and labor productivity are presented in Table 2. The other coefficients (*δ, γ*) are less important from an economic perspective because they are added mainly to the regression to improve the empirical fit of the model (Johansen and Juselius 1994). As it is common in VAR and vector error correction models, the short-run structure is specified to get well-behaved residuals.^10^

**Table 2:** Relationship between selected openness measures and labor productivity (long-run coefficient (*β*) and adjustment coefficients (*α*_1_, *α*_2_))

	(i)	(ii)	(iii)	(iv)	(v)	(vi)	(vii)	(viii)	(ix)
Liabilities, total	0.165*** (16.09)								
Liabilities, FDI		0.131*** (10.07)							
Liabilities, equity			0.134*** (10.01)						
Liabilities, debt				0.198*** (14.32)					
Trade openness					0.277 (1.09)				
Real openness						−1.771* (1.84)			
Export rate (without gold and transit trade)							0.411*** (5.46)		
Import rate (without gold and transit trade)								0.245*** (22.07)	
Real effective exchange rates									3.178*** (4.01)
Adjustment coefficient 1	− 0.26*** (2.89)	− 0.11* (1.69)	− 0.17** (2.11)	− 0.21** (2.58)	− 0.06 (1.72)	− 0.05** (2.37)	− 0.01 (0.138)	0.05 (0.23)	− 0.01 (0.90)
Adjustment coefficient 2	1.10 (1.39)	1.90** (2.61)	1.28 (1.17)	1.26* (1.70)	− 0.02 (0.16)	− 0.12* (1.98)	0.47* (1.83)	2.21** (2.34)	0.14*** (3.33)
Adj. *R*^2^ Eq. 1	0.128	0.107	0.116	0.094	0.005	0.079	− 0.059	− 0.055	− 0.044
Adj. *R*^2^ Eq. 2	0.009	0.111	− 0.021	0.049	0.026	0.123	0.072	0.138	0.206
No. of observations	40	40	40	40	40	40	30	30	40

*t* values in parentheses. ***significant at 1%; **significant at 5%; *significant at 10%

The results in Table 2 reveal a quite clear picture about the relationship between openness and productivity in Switzerland. For the financial openness indicators (i to iv), we find the expected positive relation with labor productivity. The respective coefficients are highly significant. In addition, for all financial openness indicators, the adjustment coefficients in the equation for productivity (*α*_1_) are significant. The interpretation is that productivity adjusts after a deviation from the long-run relationship. In contrast, liabilities do not adjust because the coefficient (*α*_2_) is not significant except for FDI. This indicates that liabilities except FDI are weakly exogenous to the system. The causal interpretation of this result is that capital inflows raise labor productivity in Switzerland. For example, an increase in total liability inflows in relation to GDP by 1 % is accompanied in the long run by an increase of labor productivity by 0.16%. Therefore, the reduction in foreign liabilities in equity capital after the financial crisis is one contributor to the weak productivity growth in Switzerland.

With regard to the trade openness indicators, the results are less conclusive. Nominal and real trade openness are not significantly related to productivity at the 5 % significance level. If we look at exports and imports without gold and transit trade, we find a significant positive long-run relation to productivity. However, the significance of the adjustment coefficients indicate that exports respectively imports adjust after a deviation from the long-run relationship. In this system, productivity is weakly exogenous. In addition to these openness indicators, we test the relationship between the real effective exchange rate and labor productivity and find a significant positive long-run relation. Therefore, a higher real exchange rate is accompanied with higher productivity. At first sight, a positive relation is not in line with theoretical considerations because an overvalued currency should dampen exports and therefore economic growth. However, Rodrik (2008) finds empirical evidence that this reasoning is only true for developing countries. He finds no negative relation for advanced economies. Moreover, the adjustment coefficients indicate that the real effective exchange rate adjusts after a deviation from the long-run relationship, productivity does not. Hence, an appreciation of the real effective exchange rate may be attributed to an increase in labor productivity.

### Analysis at the sectoral level

In this section, we explore the relation between foreign trade and labor productivity for selected sectors of the Swiss economy. For this analysis, we consider 12 branches of the manufacturing industry. Due to limited data availability, we use export volumes for each branch as an openness indicator. In order to obtain insights into the strength of these relations, we perform the same analysis as for the total economy. The results for the export volumes and labor productivity for these industry branches are presented in Table 3. They indicate a significant long-run relationship between exports and productivity for most of the branches. However, in most cases, we do not find a significant adjustment coefficient. Exceptions are the sectors agriculture, forestry and fishing, rubber and plastic products, and other non-metallic mineral products. In these sectors, productivity adjusts after a deviation from the long-run relationship. In the sector, electrical equipment and machinery and exports and not productivity adjust after deviations from the long-run relationship. Again, productivity is not endogenous in this system.^11^ In the paper and paper products sector, both variables adjust after a shock. Again, the overall result is that trade openness, measured by exports, is not an important source of productivity improvements.

**Table 3:** Relationship between exports and productivity for selected sectors

	Long-run coefficient (*β*)	Adjustment coefficient (*α*_1_)	Adjustment coefficient (*α*_2_)	Obs.	Adj. *R*^2^ Eq. 1	Adj. *R*^2^ Eq. 2
Agriculture, forestry, and fishing	0.123*** (4.18)	− 1.016** (2.72)	− 0.784 (1.49)	14	0.691	0.385
Textiles and apparel	− 3.592*** (3.97)	0.094* (1.80)	− 0.077 (1.53)	14	0.242	0.255
Paper and paper products	0.521** (2.06)	− 0.380** (1.98)	− 0.710*** (3.03)	14	0.189	0.472
Printing and reproduction of recorded media	0.694** (2.62)	− 0.185 (1.31)	0.446 (1.12)	14	− 0.062	0.030
Basic pharmaceutical products and pharmaceutical preparations	− 0.595*** (9.76)	− 0.019 (0.08)	0.590* (1.77)	14	0.330	0.080
Rubber and plastic products	0.783*** (7.08)	− 0.532*** (4.30)	− 0.265 (0.73)	14	0.629	0.219
Other non-metallic mineral products	1.821*** (4.40)	− 0.151*** (3.22)	0.359 (1.46)	14	0.741	− 0.009
Basic metals	3.554** (1.98)	0.099 (1.60)	0.133 (1.64)	14	0.096	0.104
Computer, watches, and clocks	0.377*** (8.93)	− 0.518 (1.30)	0.314 (0.46)	14	− 0.083	− 0.107
Electrical equipment and machinery	1.270*** (4.30)	0.142 (0.94)	0.868** (2.61)	14	− 0.157	0.287
Motor vehicles, trailers, semitrailers, and other transport equipment	− 2.439*** (3.58)	0.035 (0.70)	− 0.202*** (3.84)	14	0.148	0.545
Furniture	− 10.029*** (3.34)	0.008 (0.62)	− 0.061** (2.50)	14	− 0.251	0.296

*t* values in parentheses. ***significant at 1%; **significant at 5%; *significant at 10%

## Regulation of trade in services

While global trade in goods has been gradually liberalized, international trade in services is still heavily regulated. On the one hand, barriers to trade restrict foreign entry and thus dampen imports. On the other hand, restrictions affect competitiveness of domestic firms. Furthermore, services serve as essential links in global value chains and as input factors in the manufacturing process. Hence, trade restrictions in the services sector also have an impact on trade in goods (Nordås and Rouzet 2015).

### The OECD services trade restrictiveness index

The OECD Services Trade Restrictiveness Index (STRI) is a straightforward measure to assess a country’s trade policy in the services sector. It is based on a comprehensive regulatory database, which pools policy measures affecting trade in 18 services sectors. The index is calculated for 42 countries: 34 OECD countries as well as Brazil, China, India, Indonesia, Colombia, Latvia, Russia, and South Africa. The index is scaled between zero and one, where zero implies complete openness to trade and investment and one implies a completely closed sector. However, a score of 0.1 is already significant, and sectors with a score above 0.2 exhibit substantial trade restrictions (OECD 2014c). The STRI incorporates five policy areas: restrictions on foreign entry, restrictions to the movement of people, other discriminatory measures, barriers to competition, and regulatory transparency.

The STRI considers general core measures which are common for all sectors and additional sector-specific measures that take into account the characteristics of a respective sector. The scoring and weighting procedure is as follows. First, a certain policy measure is assigned a value of zero (not restrictive) or one (restrictive). Within the five policy areas, individual measures have the same weight. However, the policy areas themselves are weighted according to their relative importance in a specific sector (Geloso Grosso et al. 2015). Additionally, a STRI Policy Simulator is available online.^12^ This tool allows for analyzing the impact of a policy change on the STRI value. By setting the value of a specific policy measure to one, a more restrictive scenario can be simulated; by setting the value to zero, a more liberal scenario can be simulated.

It should be noted that the STRI presents a simplified illustration of trade barriers in service sectors. The computation involves the quantification of qualitative characteristics, primarily by a binary scoring system. Further, only de jure policy measures are considered, not how strictly these measures are actually enforced. The index allows for a straightforward comparison of trade restrictions between countries. For a comprehensive analysis, more detailed information—e.g., from the OECD Services Trade Restrictions Database—have to be taken into account.

### Services trade restrictiveness in Switzerland

**Fig. 13:**
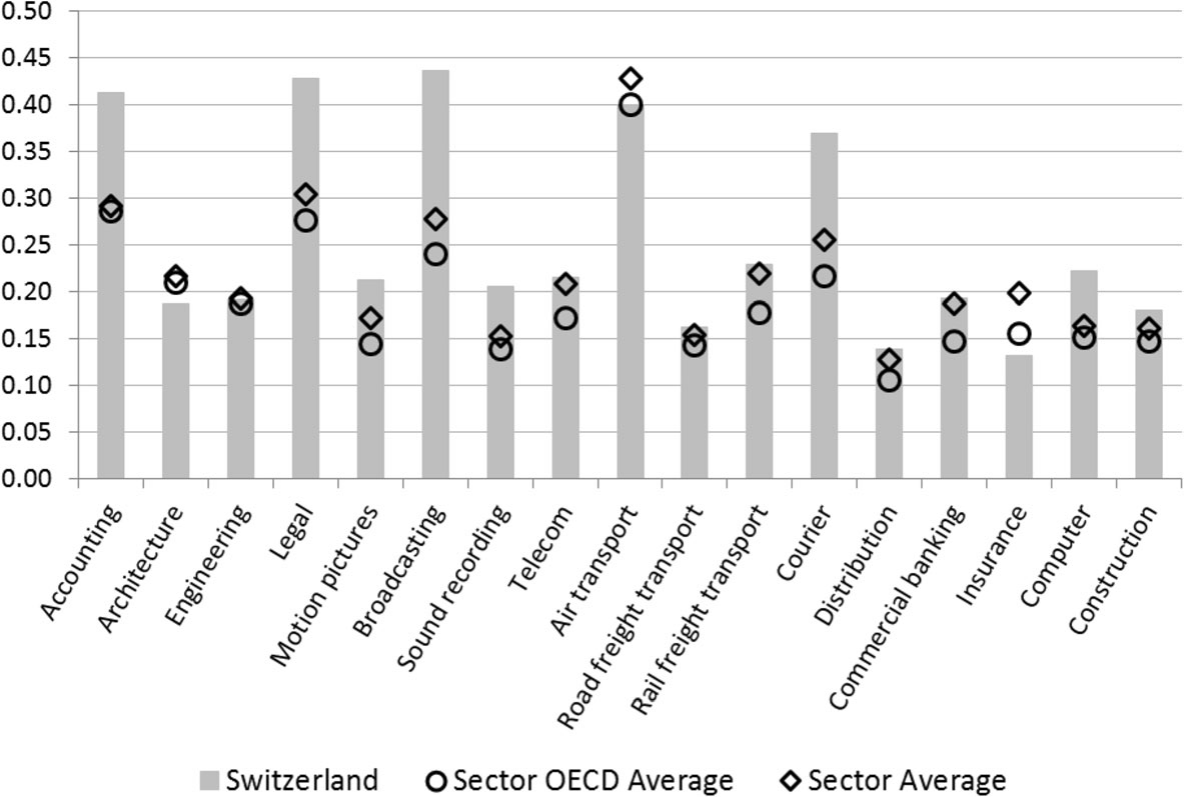
Services Trade Restrictiveness Index for Swiss service sectors. Source: OECD (2014b)

The STRI value for Switzerland is above average in 9 out of 17 services sectors (Fig. 13): legal services, accounting services, computer services, television and broadcasting, sound recording, courier services, construction, and motion pictures. The sectors with the lowest STRI value are insurance, road freight transport, and distribution. In all 17 sectors, restrictions to the movement of people contribute substantially to the STRI value. Due to trade in services being personnel intensive, regulations that constrain the movement of service suppliers impede trade in this sector.

Despite considerable restrictions to the movement of people, for nationals of an EU-25^13^ or EFTA, state regulations are less restrictive because the free-movement agreement between the European Union and Switzerland applies. This arrangement is supposed to guarantee identical living and employment conditions for foreigners and national citizens of a contracting party. The provision of services is only subject to registration as long as the stay does not exceed 90 days in a calendar year. However, stays of more than 90 days are subject to authorization by cantonal administration. Authorization is regulated by legal quotas and requires labor market tests. Additionally, foreigners have to demonstrate sufficient professional and personal qualification (State Secretariat for Migration 2015).

For non-EU/EFTA nationals, the Federal Act on Foreign Nationals (AuG) and the Decree on Admittance, Residence and Employment (VZAE) are binding. This legal basis allows for a limitation of the number of first-time short stays and residence permits for work purposes (Art. 20 AuG, Art. 19 and 20 VZAE). The admission to work in Switzerland requires that this is in the interests of the economy as a whole (Art. 18 and 19 AuG). In general, foreign non-EU/EFTA nationals may only be admitted to work in Switzerland if no suitable domestic employee or national of states with which an agreement on the free movement of workers has been concluded is available (Art. 21 AuG). Furthermore, certain personal requirements have to be fulfilled. Only managers, specialists, and other qualified workers may be admitted, provided that their qualifications and professional and social adaptability, language skills, and age give reason to expect a lasting integration into the Swiss job market and the social environment (Art. 23 AuG).

**Fig. 14:**
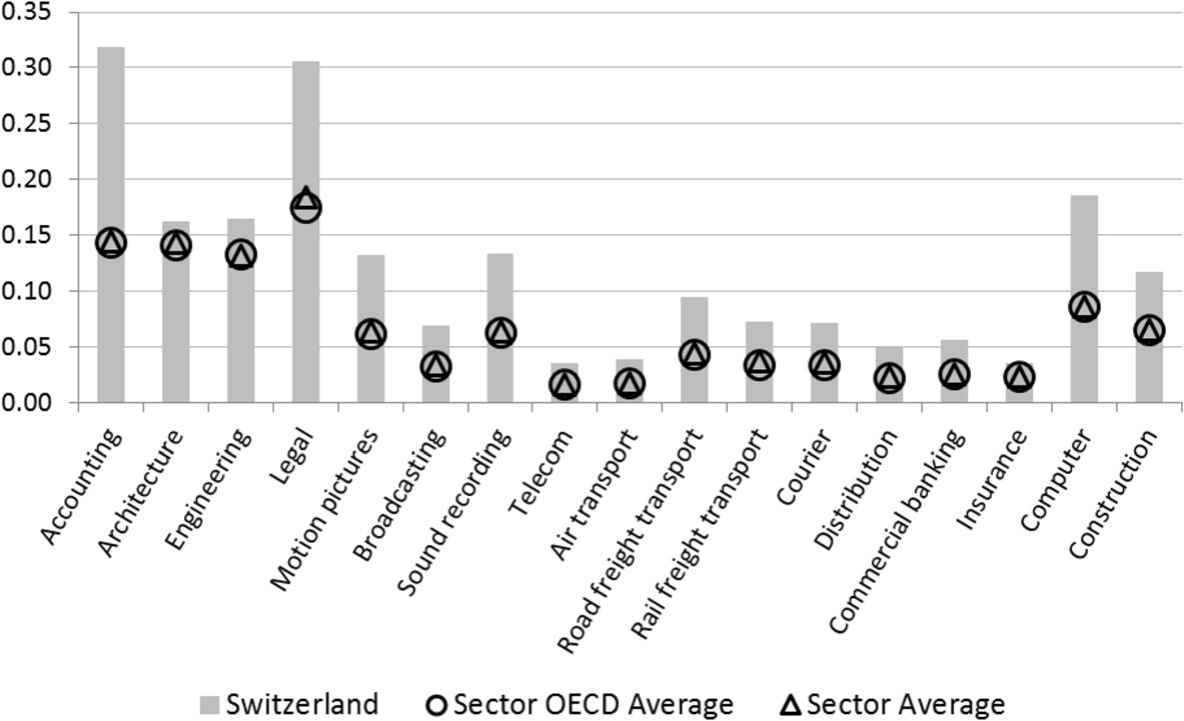
Restrictions to movement of people in the Services Trade Restrictiveness Index for Swiss service sectors. Source: OECD (2014b)

In the computation of the STRI for Swiss services sectors, the provisions specified in AuG and VZAE particularly affect the policy area restrictions to the movement of people. Three groups are distinguished in the STRI: contractual services suppliers, independent services suppliers, and intra-corporate transferees. For each of these groups, the index takes quotas and labor market test and limitations of the duration of stay into account. Accordingly, the value of the policy area restrictions to the movement of people is above average for Switzerland (Fig. 14). Further restrictions in Swiss services trade relate inter alia to the management level of corporations or the acquisition of property. For instance, at least one member of the board of directors or an executive manager of a corporation must be a resident of Switzerland (Art. 718 The Code of Obligations). Moreover, the acquisition of property or real estate by foreigners requires authorization by cantonal administration. Furthermore, the Confederation controls at least one major firm in commercial banking, broadcasting, courier services, rail freight transport, and telecommunications (OECD 2015b).

The scope of services trade restrictions captured in the STRI is markedly greater than average in the sectors legal services, accounting services, courier services, and computer services (Fig. 13). Likewise, there are considerable—and above OCED average—constraints in telecommunications and commercial banking. Moreover, while Swiss air transport scores below average, a STRI value of 0.4 indicates substantial trade restrictiveness. The barriers to trade in these branches and their impact on the STRI are explored in detail in Appendix. In the following, by means of the Policy Simulator, we will analyze potential productivity increases arising from the liberalization of trade in these sectors.

### The impact of services trade restrictiveness on trade flows and liberalization potential

Restrictive policy measures inhibit cross-border trade. On the one hand, trade barriers limit the access of foreign companies to the domestic market and thereby negatively affect imports. On the other hand, they have an impact on the competitiveness of domestic enterprises by reducing incentives for innovation and opening up new markets at home and abroad. Furthermore, services are an essential part of global supply chains. They serve as a connection between individual links in the supply chain and as inputs for the manufacturing process. Consequently, trade restrictions in service sectors may also affect trade in industrial goods (OECD 2014c).

The OECD studies the relationship between services trade restrictions and trade in services and goods (Nordås and Rouzet 2015). Their analysis is based on a gravity model which takes the Services Trade Restrictiveness Index into account and finds that barriers to trade in services sectors do not only have a negative impact on imports but affect exports as well. In computer services, legal services, air transport, maritime transport, commercial banking, and insurance, the significant negative relation between exports and STRI is even stronger than the one between imports and STRI. These results support the hypothesis that trade barriers in services sectors impede innovation in the services sector, thus obstruct international competitiveness and constrain exports by domestic service suppliers.

In this study, we design two scenarios in order to illustrate the effects of trade liberalization in services sectors on trade flows by means of the STRI policy simulator. The first scenario focuses on the liberalization of the movement of people by removing quotas for independent services suppliers, for contractual services suppliers and for intra-corporate transferees, as well as labor market tests for intra-corporate transferees. The second scenario assumes all changes made in the first scenario as well as additional sector-specific liberalizations. In legal services and accounting services, the provision that foreign providers have to completely re-do the university degree, practice, and exam in the domestic country is reversed. Further, in telecommunications, courier services, and commercial banking, the government’s control over one major firm in the sector is suspended. Moreover, in computer services and air transport, the limitation on stays for independent services suppliers, for contractual services suppliers, and for intra-corporate transferees is increased from 12 months to more than 36 months.^14^

Table 4 shows results of these policy simulations. In order to assess the impact of a change in one sector’s STRI value on the trade flows of this sector, we make use of the elasticities reported by Nordås and Rouzet (2015), who estimate the gravity model for a panel of the 40 countries that are encompassed in the STRI. To interpret the results, we assume that the trade elasticities in Switzerland correspond to the average effects of trade liberalization as measured by the index. If an estimated elasticity is insignificant, the effect on trade flows should also be insignificant and therefore is not reported here. The greatest effects of a trade liberalization emerge in the branches computer services, accounting services, and legal services. Imports in the legal sector could increase by 11.9% and exports by 9% in the second scenario. The boost in exports of computer services amounts to approx. 19%, while imports in accounting services could rise by 9.6%. While the simulated policy measures reduce the STRI value of the banking sector only by a small amount in either scenario, due to international linkages of commercial banks, the impact on imports and exports is substantial.

**Table 4:** Impact of services trade liberalization on trade flows in Switzerland

	STRI^a^		Imports in the respective sector^b^		Exports in the respective sector^b^	
	Scenario 1	Scenario 2	Scenario 1	Scenario 2	Scenario 1	Scenario 2
Accounting services	0.000	− 0.219	0.0	9.6		
Air transport	− 0.017	− 0.030	0.3	0.6	1.2	2.0
Commercial banking	− 0.025	− 0.036	2.7	3.8	4.5	6.5
Computer services	− 0.082	− 0.144			11.0	19.3
Courier services	− 0.023	− 0.033				
Legal services	− 0.076	− 0.156	5.8	11.9	4.4	9.0
Telecommunications	− 0.016	− 0.039				

Note: If estimated elasticities are insignificant, the effect on trade flows is insignificant and therefore not reported

Sources: Nordås and Rouzet (2015); Authors’ computations

^a^Values in columns 2 and 3 denote changes of the STRI value

^b^Values in columns 4 to 7 denote changes of imports, respectively exports, in percent. Calculations are based on elasticities reported in Nordås and Rouzet (2015)

In Switzerland—and in many other countries—trade in services is subject to substantially more regulation than trade in goods. Hence, trade liberalization may be a means to increase productivity in certain services sectors and thus promote economic growth. The Services Trade Restrictiveness Index by the OECD is a straightforward measure that illustrates barriers to trade in service sectors. According to this index the extent of restrictions in several Swiss services industries is larger than average, which is mainly the result of restrictions to the movement of people. Trade liberalization in accounting services, air transport, commercial banking, computer services, or legal services may increase trade flows by positively affecting imports as well as exports in the respective sectors.

## Switzerland and the cyclicality of capital flows

With respect to financial markets, Switzerland is an open economy. On the one hand, in the long run, this leads to efficiency gains due to a better allocation of resources. On the other hand, in the short run, the economy becomes vulnerable to international financial shocks. With increasing integration of international financial markets, the volatility of capital flows, in particular of gross capital flows (for which capital inflows and outflows are separately considered), has strongly increased in the recent years (Broner et al. 2013). The high volatility of capital flows is particularly destabilizing when capital flows are pro-cyclical and hence amplify domestic developments.

Based on the approach of Broner et al. (2013), this section analyses whether Swiss capital flows are pro-cyclical, estimating the equation

3 $$ {CF}_t=\alpha +\beta\ {X}_t+{\varepsilon}_t, $$

where *CF*_*t*_ are the capital flows (capital inflows (CIF), capital outflows (COD), net capital flows (net flows)), *X*_*t*_ represents the respective indicator for economic activity (year-on-year and quarter-on-quarter growth rate of GDP and the output gap), *α* is a constant and *ε*_*t*_ are the residuals of the model.^15^

The Balance of Payments Statistic of the International Monetary Fund (IMF) provides data for capital flows. Capital flows are defined as the sum of portfolio investment assets and liabilities, foreign direct investments, and other investments. The variable for economic activity is the real, seasonally adjusted GDP provided by the Federal Department of Economic Affairs. The output gap is measured by the deviation of GDP from a Hodrick-Prescott-filtered trend. The estimations are based on quarterly data, from the first quarter of 1999 to the third quarter of 2013.

Table 5 presents the results of different models that differ with respect to both left-hand-side and right-hand-side variables. The coefficients have the expected sign, namely that with increasing economic activity, more capital flows into the economy (gross capital inflows), but at the same time also, more domestic capital is invested abroad (gross capital outflows).^16^ However, the results further show that the relationship between capital flows and economic activity is rather low. Only model 2 has a significant coefficient and indicates that gross capital outflows are pro-cyclical.^17^ The behavior of domestic investors is apparently more relevant for domestic economic activity than that of foreign investors. This result might lead to the conclusion that Switzerland is less vulnerable to external shocks despite its openness. However, the results should be interpreted with caution due to the low number of observations.

**Table 5:** Relationship between the domestic economic activity and capital exports and imports

	(I)	(II)	(III)	(IV)	(V)	(VI)	(VII)	(VIII)	(IX)
	CIF	COD	Net flows	CIF	COD	Net flows	CIF	COD	Net flows
Constant	− 0.230 (− 0.810)	0.563* (1.891)	0.333 (1.143)	− 0.157 (− 0.631)	0.257 (0.955)	0.101 (0.388)	0.005 (0.025)	− 0.001 (− 0.006)	0.004 (0.017)
GDP (q-o-q)				0.287 (0.983)	− 0.474 (− 1.494)	− 0.187 (− 0.610)			
GDP (y-o-y)	0.118 (1.120)	− 0.289** (− 2.615)	− 0.171 (− 1.581)						
Output gap							0.147 (1.235)	***−*** 0.040 (− 0.305)	0.106 (0.855)
R^2^	0.022	0.107	0.042	0.017	0.038	0.007	0.026	0.002	0.013
No. of observations	59	59	59	59	59	59	59	59	59

*CIF* gross capital inflows, *COD* gross capital outflows

*t* statistics in parentheses. * and ** denote significance at the 10 and 5% level, respectively

Pro-cyclicality of capital flows is especially critical when extreme periods of capital flows occur as in the recent financial crisis. For example, a strong reduction in capital by foreign investors, a so-called sudden stop, can lead to liquidity problems in the affected economy, especially in turbulent economic times. In contrast, a strong increase in capital inflows, a surge, might lead to overheating of the economy during economic booms. If the surge in capital flows is followed by an appreciation of the domestic currency, price competitiveness of the economy could worsen. During the financial and the European Debt crisis, the Swiss franc appreciated substantially vis-à-vis the US dollar and the Euro, probably due to the safe haven effect. It was assumed that investors shifted their capital to Switzerland since it was perceived as politically stable and not directly affected by the European Debt crisis.

This hypothesis can be easily verified by identifying extreme periods of capital flows using the approach of Calvo et al. (2004). In a first step, moving averages of the respective capital flows are computed over the last four quarters and their annual change is calculated. Afterwards, the historical means and standard deviations over the last 5 years are computed. A period of pronounced changes in capital flows is identified if three conditions are fulfilled: (i) the annual change of capital flows is lower than two standard deviations of the historical mean in at least one quarter, (ii) the period starts when the annual change deviates by more than one standard deviation and ends when the deviation is less than one standard deviation from the historical mean, and (iii) the period is longer than two quarters.

We apply this method to gross capital flows and differentiate between four different kinds of periods: a strong decrease in capital inflows (sudden stop), a strong increase in capital inflows (surge), a strong decrease in capital outflows (retrenchment), and a strong increase in capital outflows (flight).

**Fig. 15:**
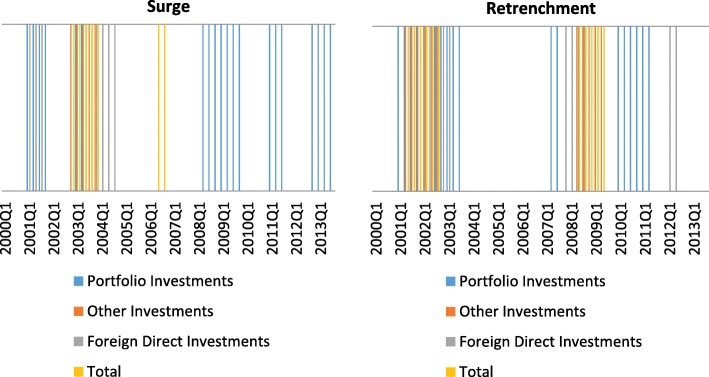
Periods of strong capital inflows (surge) and low capital outflows (retrenchments) for selected categories of capital. Periods of surge or retrenchment are indicated by a line for the particular quarter. Source: IMF. Own calculations

If Switzerland was actually assessed by investors as a safe haven during the recent financial crisis, surges should be identified during this time span. However, we only find strong increases of portfolio investment flows during this time (Fig. 15), which is in line with the results of Yesin (2013). She finds that the strong increase in portfolio investment inflows can be party explained by the issuance of liquid and safe assets by the Swiss National Bank. For all other kinds of capital flows (direct investments and other investments), Fig. 15 rather shows that domestic investors withdrew money from abroad and hence contributed to the appreciation of the domestic currency. During the recent financial crisis, this retrenchment of capital was observed in many countries, especially in advanced economies, and was an important driver of capital flows (Milesi-Ferretti and Tille (2011), Broner et al. 2013)). The observation that investors in general do not shift their capital to safe economies but rather withdraw money from abroad and invest it at home is labeled as home-bias effect. This effect is higher with increasing uncertainty (Schmidt and Zwick 2015).

In sum, the results indicate that short-run capital inflows and outflows are particularly influenced by the behavior of domestic investors. This is in line with Auer and Tille (2016) who find that Swiss banks shifted their capital to Switzerland during the recent financial and the European Debt crisis, which led to a strong appreciation of the Swiss franc. Thus, the home-bias effect was the key driver of the appreciation of the Swiss franc rather than the safe haven effect. Such developments in capital flows can be influenced by monetary policy only to a certain degree (Rey 2015), so that the disadvantages of financial openness have to be accepted in order to benefit from medium-term and long-term positive effects of financial openness on productivity.

## Conclusions

This paper analyzes the connection between trade and financial openness on the one hand and labor productivity and economic activity in Switzerland on the other. As an economy’s openness has many dimensions, we look at different definitions of openness. The overall picture emerges that the openness of the Swiss economy has increased in recent years.

The OECD, however, still labels Switzerland as a relatively closed economy (OECD, 2013b). We call this assessment into question. Employing the same methodology, we show that in terms of de facto openness, Switzerland is as open as comparable OECD countries. Differences in these two assessments result from a change in the accounting standard: We show that the treatment of transit trade, which is a very important segment of trade for Switzerland, is essential for differences in the assessment of Switzerland's openness.

We then analyze the connection between openness and productivity growth in the Swiss economy. Does further opening up the economy increase productivity? For the aggregate level, we only find weak evidence for co-movement between trade openness and labor productivity. In a second step, we employ a disaggregate analysis. For several branches of manufacturing, we find a positive correlation between real exports and labor productivity. Causality seems to run from openness to productivity. Thus, further increasing international trade in the respective branches might also increase productivity. For the services sector, the literature shows how trade deregulation fosters international trade in services (Nordås and Rouzet 2015). Using this insight, we simulate two scenarios and thereby show how policies that lower regulation in the service sector could translate into an increased international exchange of services.

With regard to financial openness, we find a positive correlation with economic activity. However, the literature argues that benefits of financial openness in the long run come at the price of higher volatility in the short run due to the volatility of financial flows. We therefore analyze the root of net capital flow volatility in Switzerland. We find that retrenchment of Swiss investments abroad, e.g., banks shifting money from international investments to domestic deposits, were the key driver of capital inflows in the aftermath of the financial crisis. Inflows of foreign investors due to the perception of Swiss being a safe haven were not the dominant factor. Short-run costs due to the high volatility of capital flows might therefore be lower than widely perceived.

## Appendix

A. Services sectors and trade restrictiveness

Legal services

Swiss legal services are assigned a STRI value of 0.428, which is significantly above the sector average (0.304) and the sector’s OECD average (0.276). This branch is in general subject to a high degree of regulation, especially regarding qualification and licensing requirements. In Switzerland, the most important provisions are specified in the Federal Act on the Free Movement of Lawyers (BGFA). Lawyers are required to obtain a lawyer’s license and to be registered in a canton in order to represent clients in court. Licenses are granted provided that a person has a graduate degree in law awarded by a Swiss university, or a similar degree from a country that grants reciprocity, and additionally has practical experience in Switzerland and has passed an examination of theoretical and practical juridical knowledge (Art. 4 and 7 BGFA).

Nationals of member states of the EU or EFTA who are entitled to practice the legal profession in their country of origin may represent clients in Swiss court under the freedom to provide services without fulfilling the aforementioned requirements, as long as the provision of services does not exceed 90 days a year (Art. 21). By registering with the cantonal supervisory authority they may permanently represent clients (Art. 27). In order to obtain the same rights and duties as Swiss lawyers, EU or EFTA lawyers have to pass an examination or demonstrate sufficient practice and expertise in Swiss law. They may then register in a canton (Art. 30). Lawyers from non-EU/EFTA member states have to acquire a Swiss lawyer’s license, by fulfilling the same requirements as Swiss nationals. However, there are no restrictions regarding the provision of consulting services by foreign lawyers in their home-country law or international law (WTO 2008).

Accordingly, for the computation of the STRI for Swiss legal services, restrictions to the movement of people are the key policy area. Moreover, it is the category carrying the greatest weight in this sector. Consequently, unilateral trade liberalization within this policy area seems suitable to significantly decrease trade restrictiveness of legal services.

Accounting services

The Swiss sector accounting services is characterized by a STRI value of 0.413, which is significantly above the sector average (0.291) and the sector’s OECD average (0.286). Accounting services comprise accounting and auditing services and book-keeping services in the STRI. There are no general restrictions on the provision of these services regarding cross-border supply, consumption abroad, or commercial presence. Auditing is, however, subject to regulation in Switzerland (World Trade Organisation 2008; OECD 2015b). Obtaining an auditing license in Switzerland requires specific education and experience. Swiss certified auditors or university graduates with at least 12 years of practical experience fulfill these requirements. Additionally, foreigners with comparable education and experience who demonstrate sufficient knowledge of Swiss law may acquire an auditing license as long as a treaty with the country of origin exists or the country holds a reciprocal right (Art. 4 Law on Audit Supervision). This is the case for member states of EU and EFTA. Moreover, auditing services to Swiss companies limited by shares have to be provided by at least one auditor who is a resident of Switzerland or has a commercial presence in Switzerland (Art. 730 The Code of Obligations).

Similar to legal services, the majority of restrictions in Swiss accounting services are found in the STRI category restrictions to the movement of people, which is ascribed the second greatest weight in this sector. In particular, the legal provisions on the recognition of auditing degrees and qualification by foreigners contribute to the high STRI value.

Telecommunications

The trade restrictiveness of the Swiss telecommunications industry as captured by the STRI (0.215) is approximately equal to the sector average, but above the sector’s OECD average (0.171). Telecommunications are characterized by market imperfections such as network externalities, access to essential facilities and switching costs which constitute a competitive advantage for incumbent firms and an entry barrier for new providers. Thus, regulation may be necessary in order to promote competition. That is why in the computation of the STRI value for this sector regulation of dominating service providers (pro-competitive regulation) is not assessed as a restrictive policy measure (Nordås et al. 2014a).

In telecommunications, the policy area barriers to competition carries the greatest weight. Furthermore, it is the category with the most essential restrictions for the Swiss sector. By law, the Confederation is the majority shareholder of the leading telecommunications company Swisscom, a former public monopoly provider (Art. 6 Telecommunications Enterprise Act). Both the fact that there is a dominant firm in all market segments (fixed, mobile, internet), and the role of the state in the sector contribute to the STRI value.

Courier services

The STRI value for Swiss courier services (0.370) is notably above the sector average (0.255) and the sector’s OCED average (0.216). Both courier services and postal services are incorporated in the STRI, categorized into letter, parcel, and express delivery. Restrictions to foreign entry is the most important policy area in the computation of the index value. For the Swiss sector, one policy measure relevant for this category is the requirement for providers of commercial postal services to have a domicile or a place of establishment in Switzerland. While restrictions to the movement of people carry the lowest weight, these regulations are the most essential ones for Swiss postal and courier services due to the general provisions on foreign nationals’ admission to work.

The policy area with the second greatest weight is barriers to competition. Similar to the case of the telecommunications industry, the Swiss Federation is the majority shareholder of Swiss Post (Art. 6 Federal Act on the Organization of Swiss Post), which is the designated postal operator in the letter market and thus the dominant provider in this segment. As laid down by law, it holds the exclusive right to deliver letters weighing up to 50 g (Art. 18 Law on the Postal System). Grosso et al. (2014) demonstrate that the existence of this statutory monopoly is the main driver for the STRI of Swiss courier services.

Air Transport

The STRI for air transport is quite high for all countries. The Swiss sector is assigned a value identical to the sector’s OECD average (0.400), and it ranks below the sector average (0.428). The industry is characterized by a multitude of sector-specific provisions, which are established by national regulations as well as bilateral air services agreements by which air traffic rights are exchanged. For instance, the Agreement between the European Community and the Swiss Confederation on Air Transport grants Swiss airlines access to the EU internal market.

Restrictions on foreign entry, other discriminatory measures, and barriers to competition are the most relevant policy areas in the computation of the air transport STRI. For the Swiss air industry, barriers to entry and competition are the most relevant restrictions. For instance, only companies that are majority-owned and controlled by Switzerland, member states of the European Union, or their nationals are granted an operating license (Art. 4(f) EC Regulation No 1008/2008). This provision contributes crucially to the score of the category restrictions on foreign entry. Furthermore, policy measures regarding the exemption of air carrier alliances from competition law and slot allocation systems are assessed as restrictive in the policy area barriers to competition. By law, air carriers are allowed to combine air services and to enter into code share arrangements (Art. 15 EC Regulation No 1008/2008). In addition, they may retain already allocated slots from one season to the next (Art. 8 EC Regulation No 793/2004).

Computer services

The STRI of Swiss computer services (0.222) ranks above the sector average (0.164) and the sector’s OECD average (0.151). Computer services as defined by the STRI incorporate computer programming, consultancy and related activities, and information service activities (OECD 2014d). The sector is competitive and there are no sector-specific regulatory measures (Nordås et al. 2014b). Restrictions to the movement of people play a vital role in this sector. While trade in computer services is facilitated by electronic networks, it also requires on-site visits for technical support or consulting services (OECD 2014d). The STRI value for Switzerland is mainly determined by the existence of quotas on the number of first-time short stay and residence permits for work purposes, labor market tests, and a limitation on the duration of stay for work purposes.

Commercial Banking

The STRI for Swiss commercial banking (0.193) is near the sector average (0.187), but still considerably above the sector’s OECD average (0.148). While the policy area barriers to trade is the one with the greatest weight in the sector index, restrictions to the movement of people is the most relevant one for Switzerland. The general regulations governing the admission of foreign nationals for work purposes apply in the commercial banking sector.

## Footnotes

1 This decrease in the index is driven by more variation in tariff rates and more restrictive capital controls. Increased capital controls seem to reflect sanctions mainly against certain individuals and African states (IMF 2005; IMF 2006). Inter alia, prescriptions on the investment of pension account and restrictions for real estate investment by foreigners (the so-called Lex Koller) are counted as restrictive capital controls.

2 See http://atlas.cid.harvard.edu/about/glossary/#economiccomplexity for information on how the index is calculated.

3 Kose et al. (2009b) find a significantly positive effect of FDI and portfolio equity liabilities on TFP growth. Alfaro et al. (2009) find that FDI can promote growth via the productivity channel, if countries are characterized by well-developed financial markets and high institutional quality, both of which can be confirmed in the case of Switzerland.

4 These demand measures have been provided by State Secretariat for Economic Affair (SECO).

5 We use the natural logarithm to transform all variables in (1).

6 For a table with the estimation results, see OECD (2013b), p. 48, (Table 1.1. Trade gravity model estimation results).

7 See also, OECD (2013b) 49 (Figure 1.1. Estimated over-performance in trade openness).

8 The general form of the error correction model we use is $\Delta {X}_t=\Pi {X}_{t-1}+\sum \limits_{i=1}^{k-1}{\Gamma}_i\Delta {X}_{t-i}+\Phi {D}_t+{\varepsilon}_t$. The lag length is selected according to the AIC. To check for cointegration, we test for the rank of the Pi matrix (which is equivalent to the number of cointegration relations). Using these restrictions, the system is estimated via maximum likelihood. The long-run coefficients are derived from the eigenvectors of the Pi matrix. The adjustment coefficients are calculated by using Π = *αβ*^′^ (Johansen 1995, 93).

9 For example, assume that there is a disruption in the long-run relation as *x*_*t* − 1_ is well above the long-run level, the coefficient *α*_1_ is significant with 0 > *α*_1_ >  − 1 and the coefficient *α*_2_ is insignificant. In such a case, the long run relation *y*_*t* − 1_ − *c*_0_ − *βx*_*t* − 1_ becomes negative. As *α*_2_ is not distinguishable from zero, *∆x*_*t*_ does not adjust to this disruption. *x* is therefore labeled as weakly exogenous to the system. Adjustment takes place via *y*. The closer *α*_1_ is to one, the quicker is the adjustment to the new long-run relationship. In the extreme case of *α*_1_ =  − 1, *∆y*_*t*_ increases one for one to the disruption and the adjustment is completed in period *t*.

10 The results of the testing procedure and for all coefficients of the model are available from the authors upon request.

11 This empirical finding is in line with the literature. Yamada (1998) does not find a robust causality running from exports to productivity in industrialized countries.

12
http://sim.oecd.org/Default.ashx?lang=En&ds=STRI

13 Austria, Belgium, Cyprus, Czech Republic, Denmark, Estonia, Finland, France, Germany, Greece, Hungary, Ireland, Italy, Latvia, Lithuania, Luxembourg, Malta, the Netherlands, Poland, Portugal, Slovakia, Slovenia, Spain, Sweden, and the UK.

14 EU regulations prevail in the air transport industry, particularly in the policy areas restrictions to foreign entry and barriers to competition. Hence, liberalization in these areas seems to be less feasible.

15 Considering a trend component in the equation as in Broner et al. (2013) does not change the results, and therefore, a trend component is ignored here.

16 Negative gross capital flows mean that more capital is invested abroad.

17 This result is in line with that of Broner et al. (2013), despite the negative coefficient. Note that gross capital flows are negatively defined in our paper, in contrast to Broner et al. (2013).
